# Prevalence of Diabetes, Hypertension, and Associated of Cardiovascular Diseases: A Comparative Pre- and Post-COVID Study

**DOI:** 10.3390/diseases12120329

**Published:** 2024-12-13

**Authors:** Manuela Chiavarini, Jacopo Dolcini, Giorgio Firmani, Elisa Ponzio, Pamela Barbadoro

**Affiliations:** 1 Department of Biomedical Sciences and Public Health, Section of Hygiene, Preventive Medicine and Public Health, Polytechnic University of the Marche Region, 60126 Ancona, Italy; 2 Department of Health Sciences, University of Florence, Viale GB Morgagni 48, 50134 Florence, Italy

**Keywords:** COVID-19, chronic diseases, diabetes, hypertension, socioeconomic determinants

## Abstract

**Background**: Diabetes and hypertension are major global health challenges aggravated by COVID-19’s impact on healthcare and lifestyle factors. This study aims to compare the prevalence and associated socio-demographic factors of these conditions before and after the pandemic (2019 vs. 2022). **Materials and Methods**: We used data from Italy’s “Aspects of Daily Life” survey; 74,294 adults were included. **Results**: Results show a rise in diabetes prevalence from 7.76% in 2019 to 8.49% in 2022 (*p* < 0.05), while hypertension did not show this. Logistic regression analysis for the years 2019 and 2022 revealed a statistically significant association between the year 2022 and increased odds of diabetes (OR = 1.08, *p* = 0.008). BMI’s role as a risk factor intensified, with higher odds ratios (ORs) for both conditions in overweight and obese individuals in 2022. For example, obesity-related ORs for diabetes increased from 2.45 (95%CI 1.73–3.47) in 2019 to 3.02 (95%CI 2.09–4.35) in 2022, and for hypertension from 2.86 (95%CI 2.28–3.58) to 3.64 (95%CI 2.87–4.61). Lower education levels also showed a greater association with hypertension risk in 2022; subjects with only middle or high school diplomas had significantly higher ORs than individuals with higher education; there was a non-significant trend in 2019. However, diabetes risk associated with lower education remained stable and significant in both years. **Conclusions**: These findings suggest that the pandemic may have increased risk factors for diabetes and hypertension, particularly BMI and educational level, compared with the literature on the increased burden of chronic diseases during COVID-19.

## 1. Introduction

Cardiovascular diseases (CVDs) represent a leading cause of mortality worldwide [1] and in Italy, accounting for about 35% of all deaths [2]. Most cardiovascular diseases can be prevented by addressing behavioral and environmental risk factors such as tobacco use, unhealthy diet and obesity, physical inactivity, and harmful use of alcohol. Diabetes and hypertension are indeed major risk factors for cardiovascular diseases (CVDs) rather than being classified as CVDs themselves. Approximately 30% of adults in Italy have high blood pressure [3], and the prevalence of diabetes is around 5–6% [4]. The COVID-19 pandemic has had profound effects on healthcare systems worldwide, causing significant disruptions in various areas. Routine health services, such as chronic disease management, saw significant declines. Non-urgent medical procedures were postponed, prioritizing COVID-19 care, leading to waiting and delayed diagnoses for other conditions [5]. The pandemic exacerbated existing health disparities, particularly affecting marginalized communities with limited access to healthcare resources [6] and an increased prevalence of CVD risk factors [7,8,9]. COVID-19 may serve as an underappreciated CVD risk modifier, including risk factors such as diabetes mellitus or arterial hypertension [10].

Emerging evidence suggests that COVID-19 indirectly influenced chronic conditions through lifestyle changes, healthcare disruptions, and socioeconomic factors. Lockdowns and restrictions led to reduced physical activity, unhealthy eating, and increased alcohol consumption, aggravating chronic disease conditions such as diabetes and hypertension [11,12]. Furthermore, healthcare disruptions have delayed routine care and the management of chronic diseases, while the economic problems of the pandemic have impacted vulnerable populations, exacerbated health inequalities, and limited access to essential services [13]. Moreover, numerous studies have highlighted how SARS-CoV-2 infection can lead to the development of long COVID-19 syndrome, which represents an additional burden in terms of healthcare outcomes for individuals who may already be affected by pre-existing conditions such as neoplasms, renal diseases, diabetes, hypertension, anxiety and depressive disorders, chronic liver diseases, ischemic heart disease, obesity, and autoimmune diseases. In fact, it has been found that COVID-19-induced inflammation interacts with these pre-existing conditions, aggravating their impact on cardiovascular health [14,15].

The COVID-19 pandemic has dramatically influenced the incidence of cardiovascular diseases both through the direct effects of the virus and the indirect consequences of delayed care. The long-term effects of delayed care may lead to an increase in CVD incidence in the post-pandemic period, particularly among those with pre-existing conditions [16]. Moreover, it has been shown that patients with complicated COVID-19 infection have a higher risk in terms of impaired cardiac function [17] as well as an increased risk of diseases involving the heart, kidney, and metabolism in terms of a substantial increase in the prevalence of cardiovascular-kidney-metabolic (CKM) syndrome [18]. Other conditions related to cardiovascular health have been linked to COVID-19 since an increased incidence of dyslipidemia during COVID-19 has been found [19].

Diabetes and hypertension were chosen as the outcome of this study because of their dual importance; both are major contributors to CVD risk [20] and are most sensitive to healthcare access and lifestyle determinants. Moreover, hypertension and diabetes are among the most frequent and mortality-related diseases in Italy in recent findings [21]. Interestingly, some evidence has shown a strong connection between the risk of poor outcomes after hospitalization for COVID-19 and the presence of these two comorbidities [22]. Additionally, some studies have highlighted, comparing the post-COVID-19 period to the pre-COVID-19 one, that the risk of hypertension increased and the number of patients with dyslipidemia doubled, suggesting that patients affected by COVID-19 are at an increased risk of developing hypertension and related disorders [23]. Some biological evidence, in terms of shared molecular pathways, has been highlighted between hypertension and diabetes pathological expressions and SARS-CoV-2 infection. The virus enters human cells mainly through the binding of the virus-associated Spike protein and the human ACE2 receptor, which is highly expressed in several districts of human organisms. This receptor is involved in complex pathways aimed at maintaining the homeostasis of blood flow, endothelial function, insulin secretion, and lower insulin resistance, as well as increasing vasodilation, thus influencing blood pressure and glycemia levels. Several drugs used for these conditions, such as Metformin for diabetes and inhibitors of the angiotensin converting enzyme commonly used for hypertension, target the ACE2 receptor and related pathways [24,25,26,27,28,29,30]. Considering all these complex interactions spanning from healthcare and lifestyle changes during the pandemic and biological connections, diseases such as diabetes and hypertension are particularly relevant in the post-pandemic context, where delays in routine care and intensified social and biological risk factors may have caused long-term health impacts.

The objective of this study is to identify differences in the prevalence of diabetes and hypertension between the pre-COVID (2019) and post-COVID (2022) periods in relation to selected health determinants.

## 2. Materials and Methods

### 2.1. Study Design

Data from the Italian National Statistics Institute’s “Aspects of Daily Life” survey editions 2019 and 2022 were analyzed [31,32]. The survey is administered each year to a representative sample of the Italian population as part of the integrated system of multipurpose surveys on families. The aim of the survey is to identify a variety of behavioral dimensions and aspects of daily life. The questionnaire is standardized, and the survey is carried out using a sequential CAWI/PAPI mixed-mode technique. The total number of subjects combined from 2019 and 2022 aged 18 years or older was 74,294, with N = 38,378 in 2019 and N = 35,916 in 2022. In this study, the populations for the surveys were defined by the Italian National Statistics Institute (ISTAT) through a stratified sampling approach applied to municipalities. Municipalities were divided into two groups: Self-Representative (SR) municipalities, comprising those with a larger demographic size, and Non-Self-Representative (NSR) municipalities, consisting of smaller ones. The NSR municipalities were further divided into strata of equal size based on their population. Within these strata, sample municipalities (two per stratum) were selected using probabilities proportional to their population size. For each selected municipality (both SR and NSR), cluster sampling was conducted at the family level. Families serving as the clusters were randomly chosen from municipal registry lists, and all members of the selected families were included in the survey. A minimum of 24 families was sampled per municipality. The families for each sample municipality were drawn from a master sample, and data were collected on all members within the selected families. The theoretical sample size at the national level was approximately 24,000 families, determined based on cost and operational considerations. To ensure effective supervision, the total number of sampled municipalities was capped at 900. The allocation of families and municipalities across regions followed a balanced approach, aiming to provide reliable estimates both nationally and within each regional domain [33].

### 2.2. Structure of the Questionnaire

The aspects of daily life questionnaires (AVQ) AVQ survey is part of an integrated system of social surveys that collects fundamental information on individual and household daily life. The survey provides information on the citizens’ habits and the problems they face in everyday life. The questionnaire is organized into thematic sections to collect information on various behavioral aspects, consumption habits, cultural activities, and service utilization. Each section is tailored to specific demographic groups, with clearly highlighted instructions directing respondents to the relevant questions based on age, gender, and previous answers. The responses are analyzed quantitatively through frequencies and percentages to evaluate population habits and behavioral trends. Ordinal scales, such as those for satisfaction or frequency, are used to calculate mean scores and assess associations with demographic and socioeconomic variables. To ensure the validity of results, the questions include detailed instructions and closed-ended response options to minimize subjective interpretation by respondents [33]. The survey is included in The National Statistic Plan, which gathers the statistical investigations necessary for the Country.

### 2.3. Statistical Analysis

The prevalence for each condition was calculated for the respective year, and the evaluation of the difference between the two years was assessed using the chi-square test. For the outcome variables (diabetes and hypertension), NA values were excluded. To evaluate the possible independent association of socioeconomic and demographic variables, logistic regressions were carried out with hypertension and diabetes as the outcome variables and age, gender, education level, economic resources availability, and BMI as exposure variables. Variables selection followed both criteria based on previous evidence and findings and both statical investigations. Variables such as BMI are an established predictor of hypertension and diabetes, as these conditions are strongly linked to body weight [34]. Similarly, level of education is often emphasized because it can influence health knowledge and access to health care, which are factors that can influence the prevalence and management of chronic conditions [35]. Regarding statical investigations, stepwise regression, using AIC and BIC criteria, was utilized to select between models and discriminate between covariates. We performed both logistic regressions with combined datasets from 2019 and 2022 to account for the year as an independent variable associated (year 2019 = 0, year 2022 = 1) and then stratified by the year itself. The results of the logistic regressions were then compared between the two years. Variables have been codified as follows:

- Education level (university degree and post-graduate education, high school diploma, Middle school diploma, elementary school diploma, or no formal education);
- Economic resources (excellent, adequate, scarce, inadequate);
- Body Mass Index (BMI) (underweight, normal weight, overweight, obese);
- Age (continuous variable);
- Gender (male, female).

The level of significance was set at 0.05. All data have been analyzed through STATA/SE 15.1 (StataCorp LLC, College Station, TX, USA).

## 3. Results

The baseline characteristics of the populations from 2019 and 2022, including age groups, are summarized in Table 1. A total of 38,378 participants were included in 2019, while 35,916 participants were included in 2022.

Regarding diabetes, the proportion of individuals with diabetes was slightly higher in 2022 (8.49%) compared to 2019 (7.76%), with a statistically significant difference (*p* < 0.001).

For hypertension, no significant differences were observed between 2019 and 2022 (*p* = 0.145). The proportion of hypertensive individuals was 23.65% in 2019 and 24.11% in 2022.

The education level of participants showed significant differences between the two years (*p* < 0.001). The proportion of individuals with a university degree or higher was slightly higher in 2022 (16.78%) compared to 2019 (15.47%). The percentage of participants with elementary school education or no formal education was notably lower in 2022 (14.54%) compared to 2019 (17.28%).

Economic resources also showed significant differences (*p* < 0.001). The proportion of participants with “excellent” economic resources remained similar between the two years. Still, those with “poor” resources saw a decrease from 30.36% in 2019 to 28.95% in 2022 and “inadequate” from 3.67% in 2019 to 3.27% in 2022.

In terms of BMI categories, significant differences were observed (*p* = 0.018). The percentage of participants classified as underweight, overweight, and obese remained relatively stable between the two years. However, the proportion of individuals with normal weight decreased slightly from 50.33% in 2019 to 49.88% in 2022.

For the age groups, the distribution showed significant shifts. Participants aged 18–44 years accounted for 33.14% of the population in both years. The proportion of participants aged 45–64 years slightly increased from 36.55% in 2019 to 37.24% in 2022, while those aged 65 years and above remained stable at 29.40% in 2019 and 30.59% in 2022.

Finally, in terms of gender, there were no significant differences in the distribution of males and females between the two years (*p* = 0.913). Males represented 47.87% of the population in 2019 and 47.83% in 2022, while females accounted for 52.13% in 2019 and 52.17% in 2022.

We then performed logistic regression to evaluate possible independent variables associated with the outcomes of interest (diabetes and hypertension). In both 2019 and 2022, several factors showed statistically significant associations with the likelihood of having diabetes and hypertension. Specifically, education level, BMI, age, and sex were key determinants. In the following paragraphs, we discuss these results grouped by the two outcomes.

### 3.1. Diabetes

The logistic regression model, which includes subjects from both 2019 and 2022, distinguishing the years (0 = 2019, 1 = 2022), reveals a significant effect of the COVID-19 year on diabetes prevalence (Table 2). Specifically, the odds of having diabetes in 2022 are slightly higher compared to 2019 (OR = 1.08, *p* = 0.008), suggesting that the pandemic may have contributed to an increased diabetes prevalence. In fact, the change in the effect size of the OR from 2019 to 2022 has shown an increase of 8%.

The analysis also confirms that lower education levels are significantly associated with higher odds of diabetes. Subjects with only a middle school diploma (OR = 1.68, *p* < 0.001) or elementary school education/no formal education (OR = 1.97, *p* < 0.001) have notably higher odds of diabetes compared to those with a university or post-graduate degree.

In terms of Body Mass Index, overweight individuals have significantly increased odds of diabetes (OR = 1.41, *p* < 0.001), and this association is even stronger among obese individuals (OR = 2.31, *p* < 0.001) relative to those with normal weight.

Age and gender are also significant predictors, with advancing age increasing the risk of diabetes (OR = 1.61, *p* < 0.001), while females have a protective effect compared to males (OR = 0.76, *p* < 0.001).

After evaluating the combined dataset to assess the overall impact of 2022, we further analyzed the association between socio-demographic and health-related variables with diabetes stratified by year (2019 and 2022) to identify potential differences in risk factors across the two time periods (Table 3).

In 2019, individuals with a high school diploma (OR = 1.42, 95% CI [1.18–1.71], *p* < 0.001), middle school education (OR = 1.75, 95% CI [1.46–2.10], *p* < 0.001), and elementary school or no formal education (OR = 2.10, 95% CI [1.74–2.52], *p* < 0.001) had a significantly higher likelihood of diabetes compared to those with a university degree. In 2022, the same trend was observed, though the effect sizes were slightly lower for all educational levels. High school diploma holders had an OR of 1.22 (95% CI [1.04–1.43], *p* = 0.016), those with middle school education had an OR of 1.62 (95% CI [1.38–1.90], *p* < 0.001), and individuals with elementary or no education had an OR of 1.88 (95% CI [1.59–2.22], *p* < 0.001). In 2019, individuals with “inadequate” economic resources were significantly more likely to develop diabetes compared to those with “excellent” resources (OR = 1.60, 95% CI [1.06–2.41], *p* = 0.026). However, this association was not observed in 2022, where the relationship between “inadequate” resources and diabetes was not statistically significant (OR = 1.06, 95% CI [0.69–1.64], *p* = 0.795). Again, a less statistical association could be due to a major effect of the pandemic situation in 2022.

In both years, no significant associations were found between other levels of economic resources (“adequate” and “poor”) and the risk of diabetes.

Body Mass Index (BMI) was also a significant predictor of diabetes in both 2019 and 2022. In 2019, diabetic persons were more likely overweight (OR = 1.43, 95% CI [1.01–2.02], *p* = 0.041) or obese (OR = 2.45, 95% CI [1.73–3.47], *p* < 0.001). This association persisted in 2022, where overweight individuals had an OR of being afflicted by diabetes of 1.93 (95% CI [1.34–2.76], *p* < 0.001), and obese individuals had an OR of 3.17 (95% CI [2.09–4.35], *p* < 0.001).

Age was a strong predictor of diabetes in both years. In 2019, for each one-unit increase in age category, the odds of having diabetes increased by a factor of 1.63 (95% CI [1.58–1.67], *p* < 0.001). This effect was slightly lower but still significant in 2022, with an OR of 1.59 (95% CI [1.55–1.64], *p* < 0.001).

Sex showed a consistent protective effect in both 2019 and 2022. In 2019, females had significantly lower odds of having diabetes compared to males (OR = 0.73, 95% CI [0.67–0.80], *p* < 0.001). This protective effect persisted in 2022, with an OR of 0.79 (95% CI [0.72–0.86], *p* < 0.001), though the magnitude of the effect was slightly reduced.

The effects of education level, BMI, age, and sex remained significant predictors of diabetes in both 2019 and 2022. However, some notable changes were observed: the effect of BMI on diabetes risk increased in 2022, particularly for those classified as obese, while the impact of education, though still significant, appeared to be less effective. The lowering of OR may represent a less protective effect of higher education due to a pandemic contest scenario, which may play a major role in the pathway of the association. In contrast, the pandemic may have a synergistic effect with BMI that may reflect an increased effect size of the odds ratio of association. Additionally, the level of economic resources showed a weaker or less significant association with the outcome risk, possibly due to the fact that during 2022, all people across nations experienced changes in their economic situations, which may have leveled out the effects of differences increasing global poverty.

### 3.2. Hypertension

The logistic regression model for hypertension, including both 2019 and 2022 subjects, shows that the COVID-19 year does not have a significant effect on hypertension (OR = 0.97, *p* = 0.124), suggesting that the pandemic period did not influence hypertension prevalence in the same way it impacted diabetes (Table 4).

Lower levels of education are associated with higher odds of hypertension. Subjects with a high school diploma (OR = 1.09, *p* = 0.010) or middle school education (OR = 1.12, *p* = 0.001) have increased odds of hypertension compared to those with university or post-graduate education.

Economic resources play a significant role in hypertension risk, with subjects reporting “scarce” (OR = 1.39, *p* < 0.001) or “inadequate” (OR = 1.67, *p* < 0.001) resources exhibiting higher odds of hypertension compared to those with “excellent” resources.

Body Mass Index is a strong predictor, with overweight (OR = 1.58, *p* < 0.001) and obese (OR = 2.51, *p* < 0.001) individuals having substantially higher odds of hypertension relative to those with normal weight. Notably, underweight individuals show a slight protective effect (OR = 0.78, *p* = 0.002).

Age remains a highly significant predictor for hypertension (OR = 1.83, *p* < 0.001), while gender exhibits a minor protective effect for females compared to males, although this association does not reach statistical significance (OR = 0.96, *p* = 0.063).

The combined dataset to assess the overall impact of 2022 on hypertension did not show a possible association of this year with an increased likelihood of hypertension risk. Anyway, we decided to further analyze the association between socio-demographic and health-related variables with hypertension stratified by year (2019 and 2022) to identify potential differences in risk factors across the two time periods (Table 5).

In 2019, education level was not a consistently significant predictor of hypertension. While individuals with middle school education (OR = 1.10, 95% CI [0.99–1.25], *p* = 0.058) were marginally more likely to have hypertension compared to those with a university degree, the effect did not reach statistical significance. Conversely, in 2022, education played a more substantial role. Individuals with middle school education were significantly more likely to have hypertension (OR = 1.15, 95% CI [1.04–1.27], *p* = 0.006), and those with a high school diploma also had a higher likelihood of hypertension (OR = 1.11, 95% CI [1.01–1.22], *p* = 0.034).

In terms of economic resources, only the “inadequate” category was statistically significant in both years. In 2019, individuals with inadequate resources had a significantly higher likelihood of hypertension (OR = 1.98, 95% CI [1.48–2.65], *p* < 0.001). This effect was somewhat reduced in 2022, though still significant, with an OR of 1.37 (95% CI [1.01–1.85], *p* = 0.044).

BMI had a significant impact on hypertension risk in both 2019 and 2022, with increasing BMI categories associated with higher odds of hypertension. In 2019, overweight individuals had an OR of 1.82 (95% CI [1.46–2.26], *p* < 0.001), while obese individuals had an OR of 2.86 (95% CI [2.28–3.58], *p* < 0.001). In 2022, these effects were even more pronounced. Overweight individuals had an OR of 2.27 (95% CI [1.80–2.85], *p* < 0.001), and obese individuals had an OR of 3.64 (95% CI [2.87–4.61], *p* < 0.001), indicating a stronger association between BMI and hypertension in 2022 compared to 2019.

Age was a highly significant predictor in both years. In 2019, the odds of hypertension increased by 1.82 for each one-unit increase in the age category (95% CI [1.78–1.85], *p* < 0.001). This effect was nearly identical in 2022, with an OR of 1.85 (95% CI [1.82–1.89], *p* < 0.001).

Sex had a protective effect against hypertension in 2022, where females had a lower risk of hypertension compared to males (OR = 0.93, 95% CI [0.88–0.99], *p* = 0.025). In 2019, however, the effect was not statistically significant (OR = 0.99, 95% CI [0.93–1.05], *p* = 0.687).

Regarding hypertension, the effects of BMI and economic resources showed some notable changes since the effect of higher BMI on hypertension risk increased in 2022, particularly for those classified as obese, while the impact of economic resources, though still significant, appeared to be less effective, particularly for poorer people. The lowering of OR may represent a less protective effect of economic resources due to a pandemic contest scenario, which may play a major role in the pathway of the association. The association between economic resources and outcome risk appeared weaker, likely because, in 2022, widespread changes in economic conditions across populations may have mitigated the impact of disparities. In contrast, as well as observed for diabetes, the pandemic may have a synergistic effect with BMI that may reflect an increased effect size of the odds ratio of association.

## 4. Discussion

This study highlights the complex relationship between the COVID-19 pandemic, social determinants of health, diabetes, and hypertension, underscoring the need for public health initiatives to address these disparities. In our study, the COVID-19 pandemic seemed to have a significant impact on the prevalence of diabetes but not hypertension in Italy. Our analysis revealed a statistically significant increase in diabetes prevalence from 7.76% in 2019 to 8.49% in 2022, while hypertension did not exhibit a statistically significant rise during the same period from 23.65% to 24.11%. While the relative increase in diabetes may appear modest, it could translate into a significant number of new cases when applied to the general population, highlighting its potential public health impact. Moreover, a deeper investigation through combined logistic regression analysis for the years 2019 and 2022 revealed a statistically significant association between the year 2022 and increased odds of diabetes (OR = 1.08, *p* = 0.008), even after adjusting for socio-demographic factors. This suggests that the COVID-19 pandemic period may have indirectly contributed to an increase in diabetes prevalence in Italy, and the effects size increase in terms of OR by a solid 8% (2019 OR = 1, 2022 OR = 1.08) can better catch the public health implication of the pandemic event in the diabetic population. Our findings are in accordance with previous studies that found significantly higher diabetes prevalence during the pandemic compared to the pre-COVID-19 phase, showing how the epidemiology of the disease may change in terms of rates of outcomes as well as public health costs [36]. Conversely, no significant association was observed between the year 2022 and hypertension (OR = 0.97, *p* = 0.124), indicating that the pandemic may have had a differential impact on these two conditions. The lack of significance for hypertension suggests that the prevalence of this condition remained relatively stable during the pandemic, which may reflect distinct pathways of influence or resilience in hypertension risk factors compared to those associated with diabetes. Particularly, healthcare system disruptions and lockdowns may have significantly reduced the availability of useful feedback from healthcare personnel and systems to patients regarding diabetes management. This includes aspects such as continuous blood glucose monitoring, adjustments to specific medications, weight control, and dietary habits [37]. These factors may have affected diabetes more acutely than hypertension, resulting in a different impact of the pandemic scenario on the two conditions. This finding is aligned with global trends indicating that the COVID-19 pandemic has adversely affected public health, contributing to an increase in diabetes cases within the Italian population [38,39]. Specifically, diabetes has emerged as an urgent public health issue, exacerbated by social and economic factors and limited access to healthcare services during lockdowns.

Regarding hypertension, this health outcome has been shown to benefit from self-measurement, provided proper training is given, without requiring constant communication with healthcare personnel or systems that can increase, paradoxically, the number of wrong hypertension diagnoses due to the white coat syndrome [40]. This could be one reason we did not observe a significant increase in hypertension, which showed stable numbers across the two years. However, it is important to note that other studies have reported opposite findings. A recent narrative review highlighted that typical factors of the pandemic, such as changes in health behaviors [41,42], stress [43], social isolation [44], increased socioeconomic disparities [45,46], and disruptions to physical activity [47], can negatively impact hypertension outcomes [48]. Age remained a significant factor associated with both diabetes and hypertension across both years. The odds ratios for age in our models indicate a progressive increase in risk for each unit rise in age category, underscoring the well-known association between age and chronic disease risk, although the risk for diabetes was slightly lower in 2022 compared to 2019. Education level also played a notable role, with individuals with lower educational attainment exhibiting higher odds of both diabetes and hypertension compared to those with a university degree or higher. This aligns with existing literature linking lower educational levels to poorer health outcomes, possibly due to factors like reduced health literacy, limited access to resources, and higher levels of stress [49].

A significant increase in the relation between overweight and obesity and the conditions of interest was observed between 2019 and 2022. In 2019, overweight people showed an OR of 1.43, already significantly higher than people with normal weight. This increased in 2022, reaching an OR of 1.93. The situation is even more serious for obese people, who in 2022 had an OR of 3.02, compared to 2.45 in 2019. These data indicate that obesity remains a critical risk factor for diabetes but that this risk is growing. The reasons for this increase could be due to the indirect effects of the COVID-19 pandemic, changes in lifestyle and diet, increased sedentary behavior, or other environmental and socio-economic factors that have had a greater impact in recent years.

In Italy, lifestyle changes, such as reduced physical activity and increased weight, have been linked to a rise in cardiovascular diseases (CVD) and diabetes [50,51,52].

Looking at the wider picture, it could be hypothesized that an exceptional public health event like a pandemic may reduce the protective effects of specific socio-demographic and economic characteristics such as age, education levels, and economic resources and, at the same time, increase the negative effects of others, like BMI. This effect could have been caused by several factors, such as increased bad habits due to social restrictions, reduced availability of healthcare resources fully concentrated on facing the pandemic, lowered adherence to prevention campaigns, and reduction of routine outpatient check-ups. Moreover, in this specific case of the COVID-19 pandemic caused by SARS-CoV-2, it has been shown that some pathological pathways influenced by the virus infection and penetration themselves can facilitate progression to cardiovascular diseases [53]. In fact, there is some evidence suggests that COVID-19 syndrome may act directly as a possible disruptor of the endothelial function that represents a key target organ in COVID-19, providing a mechanistic rationale behind possible association with several diseases that are strictly connected to an altered endothelial function such as diabetes, hypertension, kidney disease, and thromboembolism [54,55]. Interestingly, this relationship between endothelial dysfunction and COVID-19 has been studied and investigated as a possible reason for post-COVID-19 syndrome and chronic fatigue syndrome (ME/CFS9, since some patients affected by these conditions have shown endothelial dysfunction and the presence of altered endothelial biomarkers [56]. One of the main health burdens in patients suffering from long COVID-19 syndrome is represented by cardiovascular autonomic dysfunction (CVAD), and there is some proof linking this pathological condition to endothelial dysfunction due to COVID-19 infection [57]. The role of the endothelium is also testified by other emerging evidence that found the possible role of microcirculation in specific districts, such as the retinal one, as a potential marker for chronic fatigue syndrome [58]. Our study has several strengths. Firstly, it is based on a large, nationwide sample population, allowing for robust comparisons across two distinct time periods, pre- and post-pandemic. This comprehensive dataset, collected through the ISTAT “Aspects of Daily Life” survey, is grounded in rigorous statistical and methodological standards. Data collection was performed by trained personnel using a standardized protocol, ensuring consistency and reliability. Additionally, the survey’s design, covering a wide array of socio-demographic and health-related variables, allowed for a detailed examination of potential factors associated with diabetes and hypertension, providing valuable insights into the interplay between chronic conditions and the COVID-19 pandemic. All these elements may have contributed to estimating the prevalence and trends of diabetes and hypertension in our sample, which can be considered an acceptable proxy for the Italian population.

Nevertheless, several limitations must be acknowledged, as they may introduce potential biases that could influence the interpretation of our findings. First, the cross-sectional design of the study precludes any causal inference regarding the impact of the pandemic on the outcomes studied. While associations can be identified, the directionality of these relationships remains speculative. Second, the reliance on self-reported data introduces the possibility of declarative bias, particularly for variables such as height, weight (used to calculate BMI), and the presence of chronic conditions like diabetes and hypertension. This bias could result in either over- or under-estimation of prevalence rates. Third, the absence of direct medical verification for self-reported chronic conditions further limits the precision of our findings, as they are dependent on participant recall and reporting accuracy. Additionally, the potential for non-response bias exists, as participants may have chosen not to answer questions regarding their health, leading to a relatively high number of missing values (NA) for certain variables. While NA values were excluded from all analyses, this may have reduced the statistical power of our sample, potentially explaining the loss of statistical significance for some of our results. Lastly, it is important to note that the official language of ISTAT, the government institution conducting the survey, is Italian. The survey was not officially administered in other languages, which may have introduced selection bias by excluding individuals—despite being residents in Italy—who did not fully understand Italian, potentially skewing the sample's representativeness in a diverse population.

It is also important to consider the magnitude and direction of these biases. For instance, declarative bias in BMI reporting may disproportionately affect individuals in higher weight categories, leading to an underestimation of the true prevalence of obesity and its associated risks. Similarly, the reliance on self-reported diagnoses without medical confirmation could result in under-reporting among populations with limited healthcare access or awareness, further complicating the accurate assessment of these conditions' prevalence.

Nevertheless, our findings highlight a significant association between the pandemic year and diabetes, which may be attributed to indirect effects such as healthcare system disruptions, reduced access to preventive services, and lifestyle changes during lockdowns. Future longitudinal studies that incorporate lifestyle variables—such as physical activity, dietary habits, and access to healthcare services—are needed to clarify the pandemic’s role in these trends. Moreover, our survey lacked important information, such as SARS-CoV-2 infectious status. Then, we are unable to properly distinguish between the amount of likelihood of developing the outcomes studied, which is due to bad eating habits, sedentary, loss at follow-up of medical check-ups, and the amount which is due to virus infection and potential endothelial dysfunction that has been shown to increase susceptibility to cardiovascular pathologies as indicated by literature [59]. Moreover, future research could explore whether individuals infected with SARS-CoV-2 who did not have pre-existing comorbidities at the time of their initial infection are at a higher risk of developing cardiovascular conditions, such as hypertension and diabetes, in the future. If a causal relationship is confirmed, the widespread diffusion of the virus during the pandemic could potentially lead to a subsequent increase in non-communicable diseases, such as cardiovascular diseases. This understanding could help predict and, potentially, prevent a pandemic-like spread of such conditions through targeted public health interventions.

## 5. Conclusions

In light of our results, after a pandemic event that has caused a major disruption to health systems at national and international levels, the already established efforts in prevention and early detection are essential to reduce the burden of these diseases, but they need greater attention and intensity to mitigate the negative consequences of this pandemic.

In response to these trends, Initiatives like EuroHealth highlight the importance of tackling social determinants of health and reducing health disparities to lower the mortality and morbidity rates associated with CVD and diabetes across Europe. This initiative proposes to take concrete actions until 2027, with the overall objective of helping countries to achieve the WHO 2025 and Sustainable Development Goals on Non-Communicable Diseases (NCDs) [60].

Italy has implemented various public health strategies aimed at combating cardiovascular disease, including regular screening programs for hypertension and diabetes, public awareness campaigns promoting healthier lifestyles, and improving healthcare access. These initiatives are essential to address the rising prevalence of diabetes and CVD and ensure that patients receive the necessary support for managing their conditions.

## Figures and Tables

**Table 1:** Baseline characteristics.

Variable	2019 (N = 38,378)	2022 (N = 35,916)	*p*-Value
**Age class**			
18–44 years	13,069 (34.05%)	11,555 (32.17%)	0.00
45–64 years	14,027 (36.55%)	13,376 (37.24%)	
65 years and above	11,282 (29.40%)	10,985 (30.59%)	
**Diabetes**			
No	33,368 (92.24%)	31,280 (91.51%)	0.00
Yes	2806 (7.76%)	2902 (8.49%)	
**Hypertension**			
No	28,203 (76.35%)	26,418 (75.89%)	0.145
Yes	8734 (23.65%)	8393 (24.11%)	
**Education Level**			
Degree/post-degree	5938 (15.47%)	6028 (16.78%)	0.00
High School Diploma	14,263 (37.16%)	14,017 (39.03%)	
Middle School Diploma	10,841 (28.25%)	10,089 (28.09%)	
Elementary school Diploma or None	6632 (17.28%)	5222 (14.54%)	
NA	704 (1.83%)	560 (1.56%)	
**Economic resources**			
Excellent	572 (1.49%)	534 (1.49%)	0.00
Adequate	24,718 (64.41%)	23,803 (66.27%)	
Poor	11,652 (30.36%)	10,396 (28.95%)	
Inadequate	1408 (3.67%)	1175 (3.27%)	
NA	28 (0.07%)	8 (0.02%)	
**BMI**			
BMI-Underweight	1104 (2.88%)	1120 (3.12%)	0.018
BMI-Normal weight	19,317 (50.33%)	17,916 (49.88%)	
BMI-Overweight	13,676 (35.63%)	12,674 (35.29%)	
BMI-Obese	4281 (11.15%)	4206 (11.71%)	
**Gender**			
Male	18,373 (47.87%)	17,180 (47.83%)	0.913
Female	20,005 (52.13%)	18,736 (52.17%)	

NA Not Available.

**Table 2:** Logistic regression model assessing factors associated with diabetes (combined data for 2019 and 2022).

Diabetes			
Variable			
**Year**	**Odds Ratio**	**95% CI**	***p*-Value**
2019 (baseline)	1		
2022	1.08	(1.02–1.15)	0.008
**Education level**			
Degree/post-degree (baseline)	1		
High School Diploma	1.31	(1.16–1.48)	0.000
Middle School Diploma	1.68	(1.49–1.89)	0.000
Elementary School/None	1.97	(1.74–2.23)	0.000
**Economic resources**			
Excellent (baseline)	1		
Adequate	0.89	(0.69–1.16)	0.403
Poor	1.06	(0.82–1.39)	0.645
Inadequate	1.33	(0.98–1.78)	0.064
**BMI**			
Normal weight (baseline)	1		
Underweight	0.85	(0.66–1.09)	0.203
Overweight	1.41	(1.32–1.51)	0.000
Obese	2.31	(2.13–2.51)	0.000
**Age**			
age (continuous)	1.61	(1.58–1.64)	0.000
**Gender**			
Male (baseline)	1		
Female	0.76	(0.72–0.81)	0.000

**Table 3:** Comparison of logistic regression models assessing independent variables associated with diabetes outcome: 2019 vs. 2022.

Diabetes						
Year	2019			2022		
Variable	Odds Ratio	95% CI	*p*-Value	Odds Ratio	95% CI	*p*-Value
**Education level**						
Degree/post-degree (baseline)	1			1		
High School Diploma	1.42	(1.18–1.71)	0.000	1.22	(1.04–1.43)	0.016
Middle School Diploma	1.75	(1.46–2.10)	0.000	1.62	(1.38–1.90)	0.000
Elementary School/None	2.10	(1.74–2.52)	0.000	1.88	(1.59–2.22)	0.000
**Economic resources**	**2019**			**2022**		
Excellent (baseline)	1			1		
Adequate	0.88	(0.61–1.28)	0.509	0.90	(0.62–1.32)	0.602
Poor	1.03	(0.71–1.50)	0.866	1.10	(0.75–1.61)	0.632
Inadequate	1.60	(1.06–2.41)	0.026	1.06	(0.69–1.64)	0.795
**BMI**	**2019**			**2022**		
Normal weight (baseline)	1			1		
Underweight	0.98	(0.69–1.38)	0.9	0.73	(0.51–1.06)	0.090
Overweight	1.43	(1.01–2.01)	0.041	1.93	(1.34–2.76)	0.000
Obese	2.45	(1.73–3.47)	0.000	3.02	(2.09–4.35)	0.000
**Age**	**2019**			**2022**		
age (continuous)	1.63	(1.58–1.67)	0.000	1.59	(1.55–1.64)	0.000
**Gender**	**2019**			**2022**		
Male (baseline)	1			1		
Female	0.73	(0.67–0.80)	0.000	0.79	(0.73–0.86)	0.000

**Table 4:** Logistic regression model assessing factors associated with hypertension (combined data for 2019 and 2022).

Hypertension			
Variable			
**Year**	**Odds Ratio**	**95% CI**	***p*** **-Value**
2019	1		
2022	0.97	(0.93–1.15)	0.124
**Education level**			
Degree/post-degree (baseline)	1		
High School Diploma	1.10	(1.02–1.01)	0.010
Middle School Diploma	1.12	(1.05–1.17)	0.001
Elementary School/None	1.07	(1.00–1.16)	0.064
**Economic resources**			
Excellent (baseline)	1		
Adequate	1.20	(1.00–1.44)	0.053
Poor	1.39	(1.16–1.68)	0.000
Inadequate	1.67	(1.35–2.06)	0.000
**BMI**			
Normal weight (baseline)	1		
Underweight	0.78	(0.67–0.92)	0.002
Overweight	1.58	(1.51–1.65)	0.000
Obese	2.51	(2.36–2.67)	0.000
**Age**			
age (continuous)	1.83	(1.81–1.86)	0.000
**Gender**			
Male (baseline)	1 (baseline)		
Female	0.96	(0.92–1.00)	0.063

**Table 5:** Comparison of logistic regression models assessing independent variables associated with hypertension outcome: 2019 vs. 2022.

Hypertension						
Year	2019			2022		
Variable	Odds Ratio	95% CI	*p*-Value	Odds Ratio	95% CI	*p*-Value
**Education level**						
Degree/post-degree (baseline)	1			1		
High School Diploma	1.08	(0.98–1.19)	0.128	1.11	(1.01–1.22)	0.034
Middle School Diploma	1.10	(0.99–1.22)	0.058	1.15	(1.04–1.27)	0.006
Elementary School/None	1.04	(0.94–1.16)	0.431	1.11	(0.99–1.24)	0.058
**Economic resources**	**2019**			**2022**		
Excellent (baseline)	1			1		
Adequate	1.26	(0.97–1.63)	0.078	1.13	(0.87–1.47)	0.348
Poor	1.50	(1.16–1.94)	0.002	1.29	(0.99–1.68)	0.064
Inadequate	1.98	(1.48–2.65)	0.000	1.37	(1.01–1.85)	0.044
**BMI**	**2019**			**2022**		
Normal weight (baseline)	1			1		
Underweight	0.87	(0.70–1.08)	0.220	0.69	(0.55–0.87)	0.002
Overweight	1.82	(1.46–2.26)	0.000	2.27	(1.80–2.85)	0.000
Obese	2.86	(2.28–3.58)	0.000	3.64	(2.87–4.61)	0.000
**Age**	**2019**			**2022**		
age (continuous)	1.82	(1.78–1.85)	0.000	1.85	(1.82–1.89)	0.000
**Gender**	**2019**			**2022**		
Male (baseline)	1			1		
Female	0.99	(0.93–1.05)	0.687	0.93	(0.88–0.99)	0.025

## Data Availability

The data undergo anonymization and aggregation by ISTAT and are subsequently made publicly accessible for research purposes at the following link: https://www.istat.it/it/archivio/129956.
